# Clinical management of CMML—State of the art

**DOI:** 10.1111/bjh.20213

**Published:** 2025-06-24

**Authors:** K. Nachtkamp, F. Schulz, N. Gattermann, U. Germing

**Affiliations:** ^1^ Department of Hematology, Oncology and Clinical Immunology Heinrich Heine University Duesseldorf Düsseldorf Germany

**Keywords:** chronic myelomonocytic leukaemia, CPSSmol, MDS/MPN overlap, treatment of CMML

## Abstract

Chronic myelomonocytic leukaemias (CMML) are myeloid neoplasms characterized by a sustained increase in monocyte counts in the peripheral blood, accompanied by dysplasia, abnormal proliferation, chromosomal anomalies and somatic mutations of haematopoietic cells. More than 95% of CMML patients harbour somatic mutations. CMML must be separated from other myeloid neoplasms and reactive monocytosis. The clinical presentation of CMML varies, but most frequently shows signs and symptoms of haematopoietic insufficiency or myeloproliferation. Robust instruments are available for assessing the prognosis of patients with CMML, such as the CMML‐specific prognostic scoring system molecular. Treatment options for patients with CMML are still inadequate and generally less effective than those for other myeloid neoplasms. The only curative approach is allogeneic stem cell transplantation. This article explains essential aspects of CMML pathophysiology and provides an overview of diagnostic considerations, prognostic assessment and therapeutic options.

## Graphical abstract

Patients with chronic myelomonocytic leukaemia (CMML) typically present with clinical feature including splenomegaly, constitutional symptoms and autoimmune phenomena, as well as with pathological blood counts and biological features in the marrow. These characteristics may influence the assignment to prognostic categories such as the CPSSmol and also influence indications for therapy. Therefore, taking the patients' history and clinical complaints, a comprehensive analysis of blood and marrow including cytomorphology, histomorphology, flow cytometry, karyotyping and mutational analyses are mandatory to assess prognosis and indications of treatment. Treatment options for patients with CMML are still inadequate and generally less effective than those for other myeloid neoplasms. The only curative approach is allogeneic stem cell transplantation.

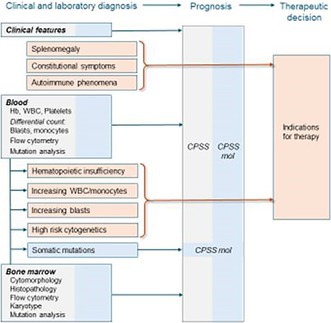

## REVIEW

Chronic myelomonocytic leukaemia (CMML) comprises a heterogeneous group of clonal bone marrow diseases that are comparatively rare: The incidence of CMML is less than 1/100 000/year but is constantly rising due to the demographic effect of the ageing of society. Precise epidemiological data are not really available, since epidemiological studies have mostly recorded CMML as a myelodysplastic syndrome, creating uncertainty regarding the exact incidence and prevalence.^1^, ^2^, ^3^, ^4^ Williams et al. provided a thoughtful discussion of this problem when they published the first well‐designed epidemiological study of CMML^5^ in this journal. Assessing the incidence of this myeloid malignancy becomes even more difficult when we consider that CMML may be preceded by a clonal monocytosis of unclear significance.^6^

The development of CMML is driven by somatic mutations in haematopoietic stem cells that can alter DNA methylation (*TET2*, *DNMT3A*, *IDH1*, *IDH2*), RNA splicing (*SRSF2*, *SF3B1*, *U2AF1*, *ZRSR2*), histone modification (*ASXL1*, *EZH2*), cell signalling (*NRAS*, *KRAS*, *CBL*, *NF1*, *PTPN11*, *JAK2*) and transcription factors (*RUNX1*, *SETBP1*, *GATA2*).^7^ *NPM1* and *FLT3* are rarely mutated but indicate potential rapid progression to acute myeloid leukemia (AML).^8^ About 95% of patients with CMML harbour one or more somatic mutations. *TET2* is mutated in about 60%, *SRSF2* in 50%, *ASXL1* in 40% and other genes in less than 15%. Combinations of mutated genes are frequent. This is in line with age‐related accumulation of somatic mutations and clonal evolution. Genes with an epigenetic function are often affected early, followed by genes involved in RNA splicing, and, later on, by genes participating in signalling pathways. CMML mutations that alter the RAS pathway are associated with myeloproliferation and transformation into acute leukaemia, *NRAS* being the most frequent. About 80% of patients with CMML show a normal karyotype, but trisomy 8, aberrations of chromosome 7 and complex karyotypes are associated with an increased risk of transformation to acute leukaemia. Deletion of 5q is a rare finding in CMML.

## DIAGNOSIS

CMML was first described in the early 1970s^9^ and has been reported as a preleukaemic state. The model of leukaemic transformation corresponds to that of myelodysplastic syndromes. However, CMML differs from myelodysplastic neoplasms (MDS) in the obligatory monocyte population that is detectable in the blood at diagnosis. Due to the similarity of CMML and MDS, the French American British classification (FAB) proposals^10^ classified CMML and MDS as close relatives, thus creating a problem because many people now believe that CMML is just a variant of MDS. However, it has become clear that CMML is more than MDS plus monocytosis, not only based on its clinical presentation but also with regard to genetic alterations and therapeutic problems. The FAB classification proposed an obligatory monocytosis of more than 1000/μL, which was retained over the years until the threshold was lowered to 500/μL in both the WHO classification of 2022^11^ and the International Consensus Classification (ICC) classification.^12^ In addition, signs of dysplasia in blood and marrow have always been included as characterizing features of CMML. Inspired by the concept of ‘oligomonocytic leukaemia’,^13^, ^14^ the threshold of 500 monocytes/μL is now used as a defining event in CMML, together with evidence of clonality by the means of detection of somatic mutations or chromosomal aberrations. This proposal solves, at least in part, another problem in the diagnosis of CMML, namely the differentiation against various non‐myeloid diseases with reactive monocytosis, as well as distinction between CMML and some other myeloid malignancies.^6^, ^15^ Table 1 presents a list of differential diagnoses that need to be excluded, especially in those rare cases without proof of clonality. The increasing availability and decreasing cost of DNA sequencing promote investigations of clonality, which either confirm the suspected diagnosis of CMML or, in case of negative findings, point to reactive monocytosis. There is evidence that molecular screening can be performed on peripheral blood cells.

**TABLE 1:** Differential diagnoses of CMML.

Malignant stem cell disorders	Non‐malignant stem cell disorders
Myelodysplastic neoplasms	Monocytosis in the context of acute bacterial infections, septicaemia
Myelodysplastic/myeloproliferative neoplasms	Monocytosis in the context of viral infections (HIV, etc.)
Myeloproliferative neoplasms (PMF, CGL)	Chronic infections (Tbc, Leishmaniosis, etc.)
Chronic eosinophilic leukaemia	Inflammatory diseases (RA, SLE, etc.)
Acute monocytic leukaemia/acute monoblastic leukaemia	Immune thrombocytopenia (ITP)
Juvenile myelomonocytic leukaemia (JMML)	Hypersplenism (splenomegaly)
VEXAS	Other reasons for monocytosis
Systemic mastocytosis	

Abbreviation: CMML, chronic myelomonocytic leukaemia.

## CLASSIFICATION

**FIGURE 1:**
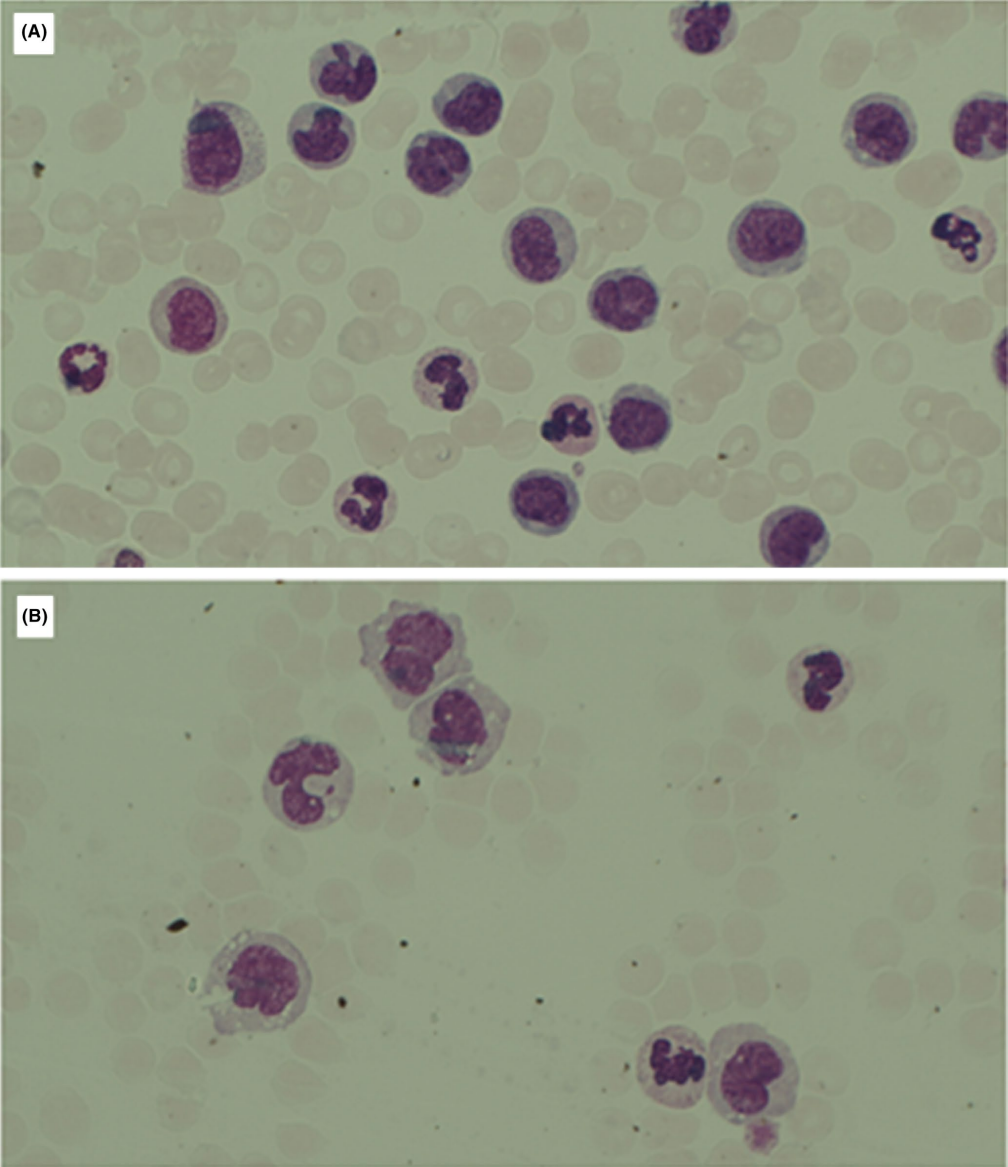
(A, B) Monocytes in peripheral blood.

**FIGURE 2:**
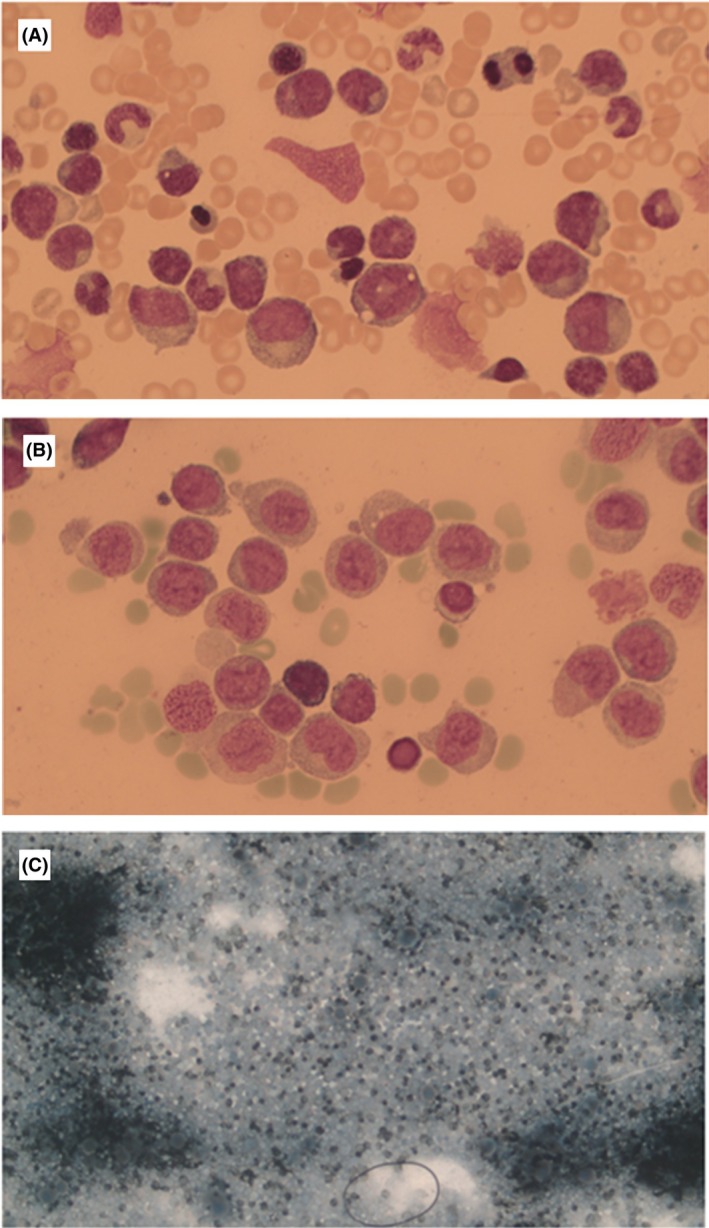
Cytology of the marrow (A) more mature monocytes, (B) promonocytes, (C) esterase staining highlighting massive monocyte infiltration in the marrow.

For good reason, both the WHO 2022 and the ICC maintain the subclassification of CMML into a dysplastic variant (CMML dysplastic) and a proliferative variant (CMML proliferative).^16^, ^17^, ^18^ Patients presenting with the proliferative variant have fewer cytopenias, higher white blood cell counts (WBCs), higher absolute monocyte counts, greater organomegaly, more pronounced lymphadenopathy, more extramedullary manifestations including skin and kidney, more frequent autoimmune phenomena including thrombocytopenia, neuropathies and, above all, more severe constitutional symptoms such as night sweats, subfebrile temperatures and signs of catabolism, findings that are typical of proliferative neoplasia such as primary myelofibrosis.^19^, ^20^, ^21^, ^22^, ^23^, ^24^ Patients with the dysplastic type of CMML generally suffer from haematopoietic insufficiency and are thus more reminiscent of a myelodysplastic syndrome. Table 2 presents the WHO 2022 classification criteria for the diagnosis of CMML (Table 2). The diagnostic parameters listed in Tables 3 and 4 are obligatory for establishing a diagnosis of CMML. The diagnosis is based on cytomorphological assessment of peripheral blood as well as cytomorphological (Figures 1 and 2) examination of the bone marrow aspirate and histopathological examination of a bone marrow threphine biopsy using immunhistochemistry. The percentage of blasts, including promonocytes, must be assessed in both blood and marrow cytology. Esterase staining in cytology may allow a better identification of monocytes, particularly it may help to distinguish monocytes from degranulated myelocytes. Signs of dysplasia should be described primarily by cytomorphology. Iron staining is used to detect ring sideroblasts. The assessment of cellularity, fibrosis and mastocytosis requires histopathological review. Although increased monocytic cells in the marrow can be detected in virtually all cases by cytomorphology and histopathology, the percentage of monocytic cells in the marrow is not a CMML‐defining feature.

**TABLE 2:** Definitions of CMML according to the WHO 2022 classification.^11^

Prerequisite criteria
Persistent absolute (<0.5 × 10^9^/L) and relative (>10%) peripheral monocytosis
Blasts percentage <20% of cells in blood and marrow. Blasts and blast equivalents include myeloblasts, monoblasts and promonocytes
Not meeting diagnostic criteria of CML and other myeloproliferative neoplasms^a^
Not meeting diagnostic criteria of myeloid/lymphatic neoplasms with tyrosine kinase fusions^b^

Supporting criteria
Dysplasia involving >1 myeloid lineages. Morphological signs of dysplasia should be present on >10% of cells of the haematopoietic lineages in the marrow
Acquired clonal cytogenetic or molecular abnormality
Abnormal partitioning of peripheral blood monocyte subsets. Classical monocytes CD14+/CD16‐ in blood should be ≥94% of the monocytic cells

Requirements for diagnosis
Prerequisite criteria must be present in all cases
If monocytosis is >1000/μL: one or more supporting criteria must be met
If monocytosis is >500/μL and <1000/μL: supporting criteria 1 and 2 must be met

Subtypes	Subgroups based on blast percentages in blood and marrow
Myelodysplastic CMML (MD‐CMML): WBC <13 000/μL	CMML 1: <5% in peripheral blood and <10% in marrow
Myeloproliferative CMML (MP‐CMML): WBC >13 000/μL	CMML 2: 5%–19% in peripheral blood, and 10%–19% in marrow

Abbreviation: CMML, chronic myelomonocytic leukaemia.

^a^ Myeloproliferative neoplasms can be associated with monocytosis at diagnosis or during the course of the disease. A documented history of MPN excludes CMML. The presence of MPN features in the marrow and/or a high burden of MPN‐associated mutations such as *JAK2*, *CALR*, *MPL* tends to support MPN with monocytosis rather than CMML.

^b^ Criteria of myeloid/lymphatic neoplasms with eosinophilia and defining gene rearrangements should be excluded in CMML cases with eosinophilia.

**TABLE 3:** Diagnostic parameters.

Diagnostic parameters
Cytomorphology of blood and marrow blood: cell counts, differential count including absolute monocyte count in blood, signs of dysplasia marrow: medullary blast count including promonocytes, monocytic cells, esterase staining (if available), iron staining^25^
Histomorphology (cellularity, dyplasia of megakaryocytes, blast count, monocytic cells, fibrosis, mast cells)^26^, ^27^, ^28^, ^29^, ^30^
Flow cytometry of blood and marrow (monocyte types CD14+/CD16‐ population, blast types^26^, ^27^)
Cytogenetics, including banding, FISH and screening for somatic mutations^31^ (exclusion of cases with *BCR::ABL1*, *PDGFR‐α* and *‐β*, *FGFR1*, *PCM1::JAK2*)
LDH, lysozyme, serum ferritin, endogenous EPO level^32^
Examination of spleen, liver, lymph nodes and skin

**TABLE 4:** Minimal gene set for screening for somatic mutations.

Minimal gene set for screening for somatic mutations	
Epigenetic regulation	*TET2* *ASXL1* *DNMT3A* *EZH2* *IDH1* *IDH2* *BCOR*
Spliceosome	*SRSF2* *U2AF1* *SF3B1* *ZRSR2*
Cell signalling	*CBL* *NRAS* *KRAS* *NF1* *JAK2*
Other genes	*RUNX1* *SETBP1* *NPM1* *FLT3* *TP53* *STAG2*

## FLOW CYTOMETRY AND GENETIC TESTING

**FIGURE 3:**
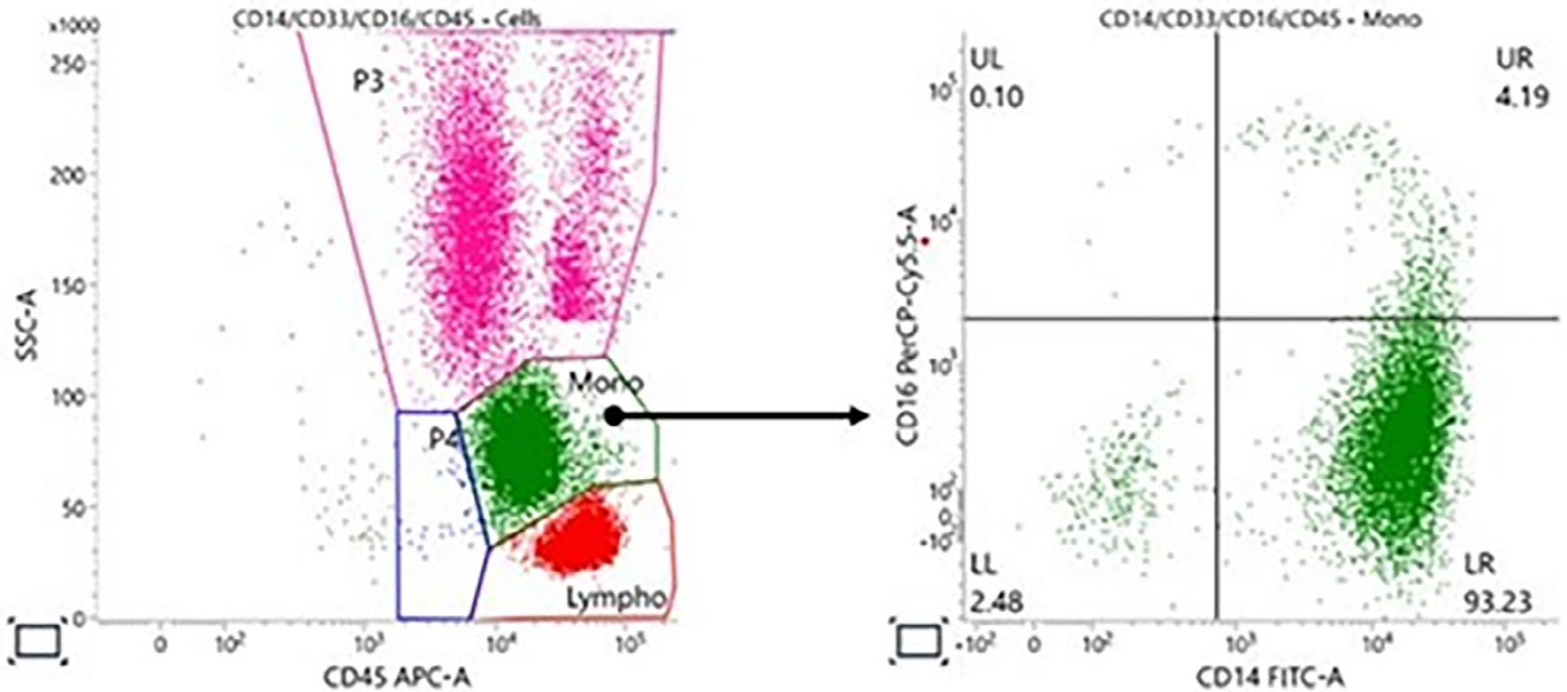
Flow cytometry of the marrow of a CMML. CMML, chronic myelomonocytic leukaemia.

Flow cytometry is used to characterize the type of monocytes in blood and marrow. In CMML, more than 95% of the monocytes are CD14+/CD16‐ cells (Figure 3). Chromosomal banding analysis of at least 20 metaphases is required and has prognostic impact. Fluorescence In Situ Hybridization (FISH) can help to detect aberrant karyotypes in cases with a low number of metaphases. Screening for somatic mutations is very important because it can provide evidence of clonality in >95% of cases and also provides prognostic information. The variant allele frequency of a somatic mutation at the time of diagnosis and its development during follow‐up can help to monitor the disease, before or during treatment, including allogeneic stem cell transplantation.

Measuring lysozyme and lactate dehydrogenase (LDH) is useful for prognostic assessment. In particular, increasing LDH values may indicate disease progression, even prior to the development of cytopenia, increasing WBCs or detection of blasts in the peripheral blood. Serum ferritin, endogenous erythropoietin levels and Human Leukocyte Antigen (HLA) typing should be considered with reference to therapeutic implications. All signs and symptoms that are compatible with reactive monocytosis should be assessed in the patient's medical history, with an emphasis on infectious and inflammatory diseases.

## CMML CATEGORIZATION

The diagnosis of CMML should eventually specify the following categories:

1. CMML proliferative versus dysplastic type
2. CMML type I versus II, according to peripheral and medullary blast counts

In addition, we recommend identifying and reporting the newly defined cases of oligomonocytic CMML, regardless of their diagnostic classification as proliferative or dysplastic type and CMML I or II respectively. In the future, this will enable us to investigate their haematological, morphological, flow cytometric, genetic and prognostic characteristics, and to find an adequate placement somewhere between classical CMML on the one hand and MDS on the other hand. It is still debatable whether a separate entity of ‘oligomonocytic chronic myelomonocytic leukaemia’ is justified.^33^, ^34^, ^35^ Similarly, there are pros and cons regarding the threshold of 5% or 10% medullary blasts as cut‐off value for CMML types.^31^, ^36^, ^37^, ^38^ Regardless, it is important to assess the blast percentage as exact and reliable as possible, at least to enable comparison between different types of disease. In addition, attention should be given to marrow fibrosis in CMML, which is present in a non‐negligible proportion of cases.^29^, ^30^ Fibrosis in CMML is associated with a worse prognosis and also with a more proliferative character of the disease, indicated by morphological and functional changes of blood cells, a more pronounced haematopoietic insufficiency, organomegaly and constitutional symptoms. The more we learn about the genetic underpinnings of myeloid neoplasms, the more genotype–phenotype correlations will be identified, obliging us to rethink classifications. There are CMML cases with AML‐type somatic mutations such as *NPM1*, *CEBPα* and *FLT3*. The question arises whether these cases should be classified as AML,^8^, ^39^ regardless of blast and monocyte counts. About 5%–10% of patients with CMML belong to the group of post‐cytotoxic myeloid neoplasia, often characterized by a poor risk type due to chromosomal or molecular events therapy‐related CMML (t‐CMML).^40^

Through assessing the above‐mentioned diagnostic parameters, important prognostic information is gathered in terms of overall survival (OS) and risk of progression to AML. In addition, at least in part, decision‐making may be alleviated regarding treatment initiation and type of therapy.

In the realm of CMML, there is another subtype of MDS/(MPN) myeloproliferative neoplasms overlap disease, namely a small group of CMML patients presenting with ring sideroblasts and/or *SF3B1* mutation.^32^ These patients formally have a CMML diagnosis but resemble the MDS/MPN *SF3B1* category with regard to clinical and haematological features and have a much better prognosis than their counterparts without *SF3B1* mutation. Apparently, the favourable prognostic impact of an *SF3B1* mutation and/or ring sideroblasts overrides the negative prognostic impact of monocytosis.

## PROGNOSIS

Prognostic assessment should go beyond the prognostic information provided by disease classifications. Table 5 lists prognostic parameters for CMML (Table 5). Neither the International Prognostic Scoring System‐revised (IPSS‐R)^41^ nor the International Prognostic Scoring System‐molecular (IPSS‐M)^42^ are well suited to identify high‐ and low‐risk CMML patients. This is because most of the parameters used in these scores are either not applicable in CMML or have a different prognostic influence in CMML versus MDS. About 80% of CMML patients present with a normal karyotype, and the majority have less than 10% blasts, a haemoglobin of more than 10 g/dL, a normal neutrophil count and platelets above 50 000/μL. Accordingly, the IPSS‐R is not particularly suitable as a prognostic tool. Regarding karyotypes, trisomy 8 is associated with a high risk in CMML but not in MDS. In summary, there was a need for CMML‐specific prognostic instruments.

**TABLE 5:** Prognostic parameters in CMML.

Peripheral blood	Bone marrow	Clinical findings	Karyotyping	Molecular findings (somatic mutations)
High monocytes^43^, ^44^, ^45^	Medullary blasts >4%,^36^, ^38^ >9%^37^	Transfusion dependency^29^, ^31^	+8, aberrations of chromosome 7, complex karyotype^29^, ^31^, ^46^	Mutation of *ASXL1* ^43^, ^47^
High leucocyte counts^31^, ^38^, ^45^, ^46^, ^47^	Marrow fibrosis^29^, ^30^	Male gender^44^		*RUNX1*, *SETBP1*, *NRAS* ^38^
Elevated LDH^48^		ECOG ≥2, MDS‐CI high^44^		*DNMT3A*, *EZH2*, *STAG2*, *TET2*, *TP53*, *U2AF1* ^49^
Immature precursors^50^		Patient care in academic centres^51^		*IDH1*, *IDH2* ^52^
Presence of blasts in peripheral blood^44^		Age^45^, ^46^		*RAS* ^53^
Lymphocytopenia^50^, ^54^				
Low Hb^29^, ^31^, ^46^, ^48^				
Low platelets <100^48^				
ANC <2500 or >16 000/μL^46^, ^55^				
Elevated serum thymidinekinase^56^				
Hypergammaglobulinaemia^57^				

Abbreviation: CMML, chronic myelomonocytic leukaemia.

### CPSS and CPSSmol

Nowadays, the CMML‐specific prognostic scoring system molecular (CPSSmol)^31^ or at least the CPSS^38^ should be applied whenever possible. The CPSS differentiates between dysplastic CMML and proliferative CMML based on WBC < vs ≥12 000/μL, separates CMML I and II by medullary blasts of </≥10%, and further considers the presence of haematopoietic insufficiency (need of regular red blood cell transfusions) and chromosomal aberrations according to three cytogenetic risk categories.^38^ The score delineates four risk categories with significantly different OS and risk of progression to AML. It was developed and validated by a Spanish–Italian–German CMML working group. A further step forward was the development and validation of the CPSSmol^31^ from the same group (Table 6). The CPSSmol in turn was a precursor to the IPSS‐M.^42^ The CPSSmol added four somatic mutations (*RUNX1*, *NRAS*, *ASXL1* and *SETBP1*) to the model, replaced the 10% cut‐off for medullary blasts with a 5% cut‐off and also defined four risk groups. The main strength of this score is the identification of a greater proportion of high‐risk patients by consideration of somatic mutations. Up to now, the prognostic influence of the variant allele frequency of somatic mutations has not been examined systematically in CMML. This task should be tackled soon.

**TABLE 6:** Definitions of the CPSSmol.^31^

Molecular risk groups					
Score	Molecular risk group	ASXL1	NRAS	RUNX1	SETBP1
0	Low	Wild type	Wild type	Wild type	Wild type
1	Intermediate	Mutated	Mutated		Mutated
2	High			Mutated	

Cytogenetic risk groups	
Score	Karyotype
0	Normal, ‐Y
1	Other anomalies
2	+8, anomalies of chr 7, complex karyotypes

Genetic score	
0	Low
1	Intermediate 1
2	Intermediate 2
≥3	High

Final combination of CPSSmol				
Score	Genetic score	Marrow blasts	White cell count	Transfusion need^a^
0	Low	<5%	<13 000/μL	No
1	Intermediate 1	≥5%	≥13 000/μL	Yes
2	Intermediate 2			
3	High			

CPSS molecular score	
Low risk	0
Intermediate 1 risk	1
Intermediate 2 risk	2–3
High risk	≥4

^a^ ≥2 red packed transfusions every 8 weeks over 4 months.

Another prognostic scoring system that works well is the Mayo Molecular Model (MMM),^43^ which includes only one mutation (*ASXL1*), but indirectly addresses inflammation by adding high monocyte counts in the blood as risk parameter. Very recently, a new prognostic score was proposed by an international working group led by Italian colleagues.^49^ In this score, which was developed with the help of artificial intelligence identifying molecular clusters associated with prognosis, conventional haematological and morphological parameters as well as chromosomal findings were combined with 10 somatic mutations (*ASXL1*, *DNMT3A*, *EZH2*, *NRAS*, *RUNX1*, *SETBP1*, *STAG2*, *TET2*, *TP53* and *U2AF1*) harbouring influence on survival and progression. This so‐called International CMML Prognostic Scoring System (iCPSS) yields five risk groups that differ significantly in terms of life expectancy and risk of AML evolution. Forty per cent of the patients were reassigned to higher or lower risk classes by the iCPSS as compared to CPSSmol. In addition, the score was applied to 753 patients who underwent allogeneic grafting and was able to stratify the probability of overall survival (OS) post‐hematopoietic stem cell transplantation. The score identified groups of patients with different probabilities of disease relapse, ranging from 9% to 62%.

### Traditional scores

A few older scores have also proven their worth. In cases without genetic information, the Bournemouth modified score, developed with a special focus on CMML patients and published in this journal in 1988,^55^ as well as the Düsseldorf score^48^ can be applied. Both scores robustly distinguish between high‐risk and low‐risk patients, allowing physicians to either embark on a treatment attempt or justify a watch and wait strategy.

In general, patients with CMML have a decreased life expectancy. Median survival of low‐risk patients is nearly the same as that in the age‐adapted normal population, while high‐risk patients have a median life expectancy of about 1 year, with a cumulative risk of about 80% for progression to AML. Overall, AML development occurs in about 20% of CMML patients, and the expected median OS time after transformation into AML is about 6 months.

It has not been thoroughly examined whether patients with oligomonocytic CMML have a more favourable outcome as compared to ‘conventional’ CMML. However, it has turned out that monocytosis in patients with MDS is associated with a worse prognosis.^58^ Altogether, a chronically elevated number of monocytes in the peripheral blood does not bode well for the patient.

## THERAPY

Considerations regarding CMML treatment are complicated for several reasons:

1. All therapies used for diverse myeloid neoplasms are less effective in patients with CMML. This is true for palliative treatment, disease‐modifying therapies and allogeneic stem cell transplantation. The probability of a treatment response and the duration of responses are worse than in AML or MDS. In CMML, there are no genetically favourable parameters that are associated with a better outcome, like t(8;21), inv(16) or *NPM1* mutations in AML. Reliable predictive parameters for predicting a response or duration of response are largely missing.
2. There is no solid basis for decision‐making regarding therapeutic goals, in particular whether we should strive to achieve normalization of peripheral cell counts or can limit our endeavour to prevent excessive numbers of leucocytes and monocytes.^59^ Furthermore, it is unclear if achieving a complete remission (in blood and bone marrow) is necessary, or if the patients also benefit from a partial response, as it is the case in patients with MDS treated with hypomethylating agents (HMA).
3. Very few compounds have been investigated in clinical trials in patients with CMML. There are only two phase 3 studies that specifically focused on patients with CMML.^60^, ^61^ Usually, patients with CMML were included in trials designed for patients with MDS. In most cases, the number of participating CMML patients was too small to be analysed separately. This was, for instance, the case in the pivotal phase 3 trial with 5‐azacitidine for patients with higher risk MDS (including CMML). Astonishingly, 5‐azacitidine was nevertheless approved for treatment of patients with high‐risk CMML of the dysplastic type.
4. 5‐azacitidine is the only approved compound for patients with high‐risk CMML of the dysplastic type (WBC <12 000/μL) in the European Union (in the US decitabine is also approved). This group comprises less than 25% of all CMML patients. Furthermore, the AZA01 study^62^ inclusion criteria categorized CMML patients into risk groups using the IPSS, which, as discussed above, is poorly suited for the prognostic assessment of CMML. Meanwhile, It has been accepted that CMML should be separated from MDS when clinical trials are designed. Accordingly, virtually all MDS studies performed in recent years excluded CMML, and specific clinical trials for CMML patients are largely lacking. It is therefore not surprising that, besides 5‐azacitidine, no other compound is formally approved for patients with CMML.
5. Although we now have robust prognostic scoring systems such as the CPSSmol that enable risk assessment in terms of disease progression and life expectancy, we should be aware that treatment is often needed for patients categorized as low‐risk CMML, with a low risk of AML development. While prognostic scores help us to assess the natural course of the disease, simple allocation of patients to various risk groups is not sufficient as a basis for therapeutic decision‐making.

**FIGURE 4:**
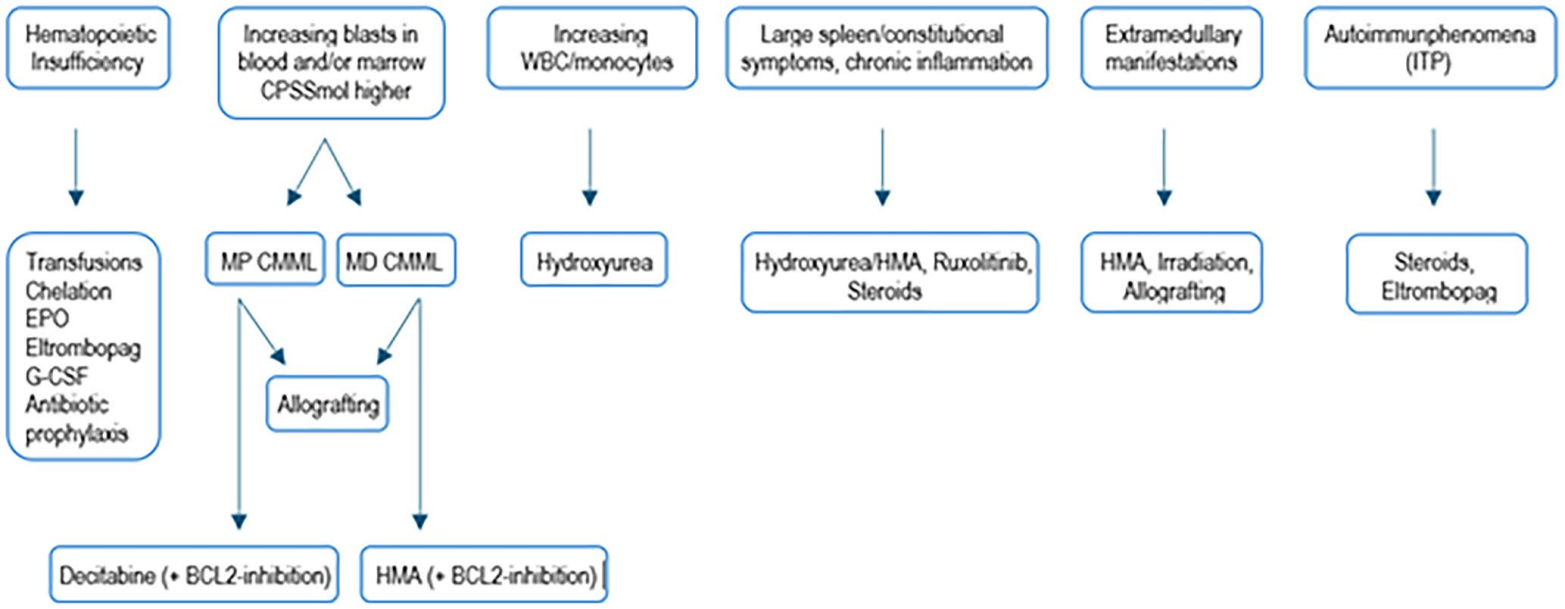
Therapeutic options in CMML according to clinical manifestations. CMML, chronic myelomonocytic leukaemia.

A reasonable strategy for avoiding unnecessary treatment is to watch and wait as long as cell counts are stable without haematological insufficiency, the patient feels comfortable and the case belongs to a low‐risk group. Due to stable disease, about 10% of CMML patients never receive treatment for their bone marrow disorder. Figure 4 presents an overview of therapeutic options with respect to certain clinical implications.

Which are the clinical scenarios that constitute an indication for treatment?

- Haematopoietic insufficiency, in particular severe anaemia (Hb <10 g/dL), low platelet counts with signs of bleeding (platelets <50 000/μL) and neutropenia (<800/μL) with increased risk of infections. These findings are mostly encountered in the dysplastic type of CMML.
- Increasing peripheral and/or medullary blast counts (>5%), indicating progression into acute leukaemia.
- Increasing white cell counts (>30 000/μL) in the peripheral blood, indicating increased proliferation of immature precursors, potentially leading to hyperleucocytosis and its complications.
- Development of splenomegaly (>5 cm below costal margin) and constitutional symptoms.
- Clinical signs and symptoms of different types of inflammation, including non‐infectious lung disease, pleural and/or pericardial effusion, ascites, joint pain and others; sometimes co‐occurrence of CMML and Vacuoles, E1 enzyme, X‐linked, Autoinflammatory, and Somatic (VEXAS).
- Extramedullary disease, such as skin infiltration or lymphadenopathy.
- Autoimmune phenomena, such as immune thrombocytopenia.

Which genetic features suggest a need for treatment?

- Clonal expansion with increasing proportion of aberrant metaphases and/or increasing variant allele frequency of mutated genes.
- Clonal evolution with evidence of additional somatic mutations or chromosomal aberrations.

### Haematopoietic insufficiency

Patients who develop haematopoietic insufficiency are generally treated like patients with myelodysplastic syndromes. Clinically relevant anaemia responds to epoetin‐alpha if the endogenous erythropoietin level is relatively low.^63^ If there is no response or a worsening of anaemia during treatment, RBC transfusions together with iron chelation can be administered according to the patient's individual needs. Data supporting other treatments for anaemia, like lenalidomide, luspatercept, imetelstat and anti‐thymocyte globulin, are largely lacking for the CMML patient population. Low platelet counts with signs of bleeding may be treated with eltrombopag and severe neutropenia can be addressed with short‐term use of Granulocyte colony‐stimulating factor (G‐CSF) in case of infections. There is no data on the long‐term prophylactic use of G‐CSF, neither in MDS nor in CMML. None of the above‐mentioned compounds are approved for patients with CMML.

In cases with somatic mutation, follow‐up of molecular findings in the peripheral blood is recommended to detect clonal expansion and/or evolution, thus identifying patients at risk of progression to a more unfavourable CMML type or AML.

### Increasing peripheral and/or medullary blast counts, indicating the presence of high‐risk CMML or impending development of acute leukaemia

In this scenario, disease‐modifying therapy and/or allogeneic stem cell transplantation should be used if possible. The situation requires discriminating between myelodysplastic and myeloproliferative CMML for different reasons: Patients with myelodysplastic CMML (MD‐CMML) can be treated with 5‐azacitidine in the EU, and with 5‐azacitidine or decitabine in the US and other countries, although this therapy is mainly palliative. Overall survival with HMA treatment is slightly prolonged, but the chances to achieve sustained complete remissions are very low. Patients with myeloproliferative CMML (MP‐CMML) respond even less frequently, and there is neither convincing evidence nor approval for HMA treatment in those patients. A phase 3 trial of a French‐German CMML group could not demonstrate that decitabine plus hydroxyurea (hydroxycarbamide) was better than hydroxyurea alone in terms of OS, even though the response rate was higher in the decitabine group.^60^ Treatment with HMA, potentially in combination with *bcl2* inhibition, must be regarded as standard treatment for patients who are at high risk to develop AML (CPSSmol high). However, the results are worse when compared to patients with MDS, as the remission rates are lower, the remission duration is shorter and the relapse rate is very high. There are no robust data indicating that HMA + *bcl2* inhibition is superior to HMA alone yet. The response rate is higher with the combination and CMML‐specific trials are on the way.^64^ A very large retrospective multicentre analysis showed that HMA led to improvement of prognosis as compared to Hydroxyurea (HU) and other low‐dose chemotherapy in high‐risk CMML types.^65^ Our knowledge of predictive markers for treatment with hypomethylating agents is poor, particularly in CMML. In the entire group of MDS patients receiving HMAs, only about 50% achieve some kind of response. Mutations of *TET2* are the only parameter that potentially predicts treatment outcome after decitabine, but this marker is not available in many places.^66^

The following things should be taken into account when considering a treatment with hypomethylating agents:

1. Before HMA treatment starts, it should be discussed with the patient if an allogeneic stem cell transplantation is possible and is the wish of the patient. It is not a good idea to treat with HMA for a long period and decide to perform an allogeneic stem cell transplantation in case of relapse or non‐response.
2. In cases with a proliferative type of high‐risk CMML, a cytoreduction could be necessary before HMA treatment. Decitabine has a higher potential to lead to normalization of WBC and can, if necessary, be combined with HU.

Unfortunately, increased medullary blasts and high WBCs are not only poor prognostic factors for untreated CMML but also predict an unfavourable outcome after treatment with 5‐azacytidine. In other words, the neediest patients benefit the least. Nevertheless, treatment with a hypomethylating agent is worth trying in CMML patients. In patients who fail to respond to first line HMA, it is justify to add the *bcl2* inhibitor venetoclax to HMA, trying to achieve a remission, taking into account prolonged and intensified aplasia.

In patients who transformed to acute leukaemia, the therapeutic dilemma is even higher. Not only the probability of response to HMA or HMA plus venetoclax is lower but also the probability of a long‐lasting remission after allogeneic transplantation is poorer.

### Induction chemotherapy

Induction chemotherapy, regardless of which combination (7 + 3 ± x or CPX351) was used, cannot be recommended for the treatment of CMML.^67^, ^68^ In case of urgent need of treatment induction, it might be considered as bridging to allogeneic stem cell transplantation.

### Allogeneic stem cell transplantation

Allogeneic stem cell transplantation is the only curative approach for CMML. However, the long‐term outcome is worse compared to myelodysplastic syndromes, primarily due to a higher relapse rate.^69^ Nevertheless, it is justified to perform allogeneic stem cell transplantation in selected patients. Comprehensive recommendations have been put together by an international expert group summing up what is known in the field.^70^ A careful ‘holistic’ assessment of patient fitness and prognosis is mandatory to select those patients who can tolerate the transplantation and to meet the challenges in the post‐transplantation period. Age and comorbidities reflected by different scores as well as the wish of the patient must be taken into account. Regarding disease biology, high‐risk disease according to CPSSmol should prompt transplantation planning without delay. Early transplantation should also be considered if a CPSSmol ‘intermediate 2’ risk is accompanied by additional risk factors such as high medullary and/or peripheral blast count, symptomatic haematopoietic insufficiency leading to transfusion need, constitutional symptoms, presence of high‐risk somatic mutations that are not included in the CPSSmol, a considerable number of somatic mutations and relapse after or refractoriness to prior therapy. CPSSmol ‘intermediate 1’ and ‘low‐risk’ disease should entail a regular reassessment of disease biology in order to detect progression as early as possible, potentially considering transplantation during the course of the disease. It is debatable if pretransplantation debulking using induction chemotherapy or HMA can improve the outcome.^71^, ^72^, ^73^ Debulking may be necessary if the malignant cells are highly proliferative, and in patients for whom a donor is not easily available. If a timely transplantation with a suitable donor is possible, pretransplant treatment is not recommended, in order to avoid selection of therapy‐resistant CMML clones. The type of donor (matched related, matched unrelated, haploidentical donor or unrelated cord blood) bears no detectable impact on treatment success, neither is the stem cell source.^74^ The choice of conditioning intensity should be based on individual circumstances, taking into account disease biology, patient fitness and donor availability. Predictive factors on OS and leukaemia‐free survival after allogeneic stem cell transplantation have been investigated in a multicentre study by Zhou et al.^75^ The authors discovered high marrow blast count (>10%), higher age (>60 years), low haemoglobin (<10 g/dL) and non‐*TET2* mutations as being independently associated with poor outcome. These results are not surprising as they represent CMML2, haematopoietic insufficiency, poor genetics and older age as universal high‐risk parameters in the context of myeloid malignancies. This means that exactly those parameters that identify a patient to be at high risk are the same that identify the patient to have a worse chance of cure.

### Increasing WBC and hyperleucocytosis

In this situation, it is necessary to find out whether leucocytosis is due to the appearance of numerous immature blasts in the peripheral blood or can be explained by an increase in monocytes and mature granulocytic cells. In the first case, very cautious cytoreduction is required, trying to avoid tumour lysis syndrome. Cell apheresis is possible but is insufficient when not paralleled by cytoreduction. Stabilization of the coagulation system may be necessary, too. If leucocytosis is due to monocytosis and neutrophilia, cytoreduction is also needed to avoid thromboembolic complications and inflammation. However, there is usually more time for cytoreduction, and the risk of tumour lysis syndrome is smaller. In that situation, the preferred treatment is hydroxyurea. Patience is needed while waiting for the cell counts to drop. Treatment must be reduced or even discontinued before normal leucocyte counts are reached. In a phase 3 study comparing hydroxyurea with oral etoposide,^61^ hydroxyurea was superior in terms of cytoreductive potency and usability. Low‐dose cytarabine can also be employed, either subcutaneously or intravenously, and may hasten the reduction of WBCs. Here again, the dosage should be reduced or treatment paused as early as possible in order to avoid long‐term cytopenia, particularly regarding platelets. Combinations with cladribine may be effective as well.^75^

### Development of organomegaly and constitutional symptoms

In this scenario, the focus is on quality of life. Constitutional symptoms, especially night sweats, fever, malaise, constipation and weight loss, are sooner or later accompanied by catabolism. Probatory treatment with a *JAK2* inhibitor like ruxolitinib^76^ is reasonable because swift improvement of symptoms may occur. However, thrombocytopenia and neutropenia must be anticipated as potential side effects. Hydroxyurea and other low‐dose cytoreductive compounds are generally not effective. Constitutional symptoms are usually accompanied by haematopoietic insufficiency and sometimes by extramedullary haematopoiesis. Treatment with hydroxyurea, steroids and HMAs is justified, but the chance of long‐term response is low.

### Clinical signs of inflammation

Patients who are symptomatic with certain types of inflammation, including non‐infectious lung disease, pleural and/or pericardial effusion, ascites, joint pain and others, can be treated with steroids. An attempt with ruxolitinib is justified, but in high‐risk CMML, HMAs may also lead to a quick response, either through suppression of the monocytic clone or through non‐directional immunosuppressive effects.

### Extramedullary disease

Extramedullary disease, particularly skin infiltration, is often not responsive to cytoreduction with HU or other compounds but may respond to decitabine or 5‐azacytidine. It often precedes progression to high‐risk CMML or even transformation to AML. Tagraxofusp, a recombinant fusion protein consisting of Interleukin‐3 (IL‐3) fused to diphtheria toxin, binds to the IL‐3 receptor (CD123) and thereby gains entrance into cells, where it blocks protein synthesis and triggers apoptosis. This drug is approved for the treatment of blastic plasmacytoid dendritic cell neoplasm (BPDCN) but may also lead to responses in cases of cutaneous manifestation of CMML.^77^

### Development of autoimmune disorders

Autoimmune phenomena are not rare in CMML. Immune thrombocytopenia, for instance, can be treated with a thrombopoietin (TPO) analogue, addressing both the increased demand on platelet production posed by the immune thrombocytopenia (ITP) and the platelet production deficit directly related to dysmegakaryopoiesis as a feature of CMML. Discussions with health insurance agencies regarding the missing approval of TPO agonists in the context of CMML can benefit from referring to the frequent occurrence of immune thrombocytopenia in CMML. Besides TPO agonists, corticosteroids and high‐dose intravenous immunoglobulins play a role in treating ITP in patients with CMML.

### Which genetic features suggest a need for treatment?

Clonal expansion and/or clonal evolution should be regarded as markers of disease progression, moving patients to a higher risk group. If clonal expansion and/or clonal evolution occurs without an increase in blasts, pre‐emptive treatment is justified to prevent progression to AML, using allogeneic stem cell transplantation. If allogeneic stem cell transplantation is not possible, HMAs can be tried. Usually, however, clonal expansion and/or clonal evolution may be accompanied by increasing blasts in the blood and marrow or dropping cell counts, triggering the same treatment decision. A standard monitoring of the variant allele frequency of mutated genes from blood or marrow is not well established in clinical routine practice outside academic centres, but can provide information on the evolution of the affected clone(s), and can indicate the necessity of treatment.

In summary, the choice of treatment for CMML patients must consider that (a) there are no approved compounds besides HMAs, (b) only a minority of CMML patients can be offered allogeneic stem cell transplantation, (c) there are no reliable predictive parameters for different types of treatment, (d) clinical trials are lacking and (e) the less favourable the CMML risk group, the greater the need for effective treatment but the poorer the results. Figure 1 could be helpful in structuring the therapeutic considerations.

### Future developments in the treatment of CMML

Proposals have been put forward on how to design future clinical trials addressing the clinical heterogeneity of CMML patients.^78^, ^79^, ^80^, ^81^, ^82^ Factors to be considered are the disease biology, as reflected by the CPSSmol risk groups, the prolonged periods without treatment indication between diagnosis and development of clinical problems and the marked heterogeneity regarding patients' suitability for intensive treatment. As the myeloproliferative type of CMML is also characterized by hypersensitivity of haematopoietic stem and progenitor cells to Granulocyte‐macrophage colony‐stimulating factor (GM‐CSF), it has been considered to use this receptor as a therapeutic target. Lenzilumab has been shown to neutralize GM‐CSF and thereby hindering an expansion of monocytes in peripheral blood and marrow.^81^, ^83^ This compound has been tested in combination with hypomethylating agents and could be beneficial for the patients. Ruxolitinib could address this pathway, too.

Another promising compound is IO‐202, an anti‐(*LILRB4*) Leukocyte immunoglobulin‐like receptor subfamily B member 4 monoclonal antibody, binding to the leucocyte immunoglobulin‐like receptor subfamily B member 4 encoded by the *LILRB4* gene at the long arm of chromosome 19q. *LILRB4* is expressed on monocytic cells and supports cell infiltration. Neutralizing this effect could be beneficial for patients.^84^ A purely oral treatment, namely the combination of oral decitabine and venetoclax has been studied in patients with MDS or CMML and could potentially established as an easy‐to use treatment in outpatient settings.^85^ Targeting *IDH1*, *IDH2* and *FLT3* in principle could be helpful in CMML, but these molecular characteristics are very unfrequent and studies have not been carried out. Treatment options to be evaluated include farnesyltransferase inhibitors like tipifarnib, compounds addressing the *RAS* signalling pathway such as onvansertib, HDAC inhibitors, *PARP* inhibitors, *JAK1* and *2* inhibitors and CDA inhibitors. There is a large unmet medical need, especially for patients who cannot benefit from allogeneic stem cell transplantation.

## AUTHOR CONTRIBUTIONS

KN and UG wrote the first draft, NG and FS revised the manuscript and provided figures and tables. All authors approved the final manuscript.
